# Long-term outcomes of suprasternal aortopexy in paediatric patients with tracheomalacia

**DOI:** 10.1007/s00383-026-06461-z

**Published:** 2026-05-04

**Authors:** Ayesha Unadkat, Iain Yardley

**Affiliations:** 1 Faculty of Life Science and Medicine, King’s College London, Guy’s Campus, Great Maze Pond, London, SE1 1UL UK; 2 Department of Paediatric Surgery, Department of Neonatology, Evelina London Children’s Hospital, London, UK

**Keywords:** Tracheomalacia, Aortopexy, Suprasternal incision, Long-term outcomes, Oesophageal atresia, Tracheoesophageal fistula

## Abstract

**Purpose:**

Tracheomalacia is characterised by dynamic tracheal collapse causing airway obstruction, particularly during expiration. In severe cases, surgical intervention with aortopexy may be required. This study reports long-term outcomes following suprasternal aortopexy, exploring predictors of operative success/failure.

**Methods:**

All patients undergoing aortopexy between February 2016 and May 2019 were included. Electronic patient records were reviewed for demographics, associated conditions, preoperative symptoms, dynamic flexible bronchoscopy findings and postoperative outcomes. Outcomes were categorised by degree of symptom improvement or resolution.

**Results:**

Twenty-eight patients (22 male, 78%) underwent aortopexy at a median age of 8 months (range 19 days–5 years 9 months) with follow-up of 6 years 7 months (range 5 years 2 months–8 years 9 months). Patients were stratified into oesophageal atresia/tracheoesophageal fistula, prematurity, syndromic and primary tracheomalacia groups. Short-term improvement occurred in 75%, with complete resolution in 46%. At long-term review, 82% improved and 64% were symptom-free. Respiratory infection was the most common persistent symptom. Syndromic patients had the poorest outcomes and no later improvement.

**Conclusions:**

Suprasternal aortopexy is a safe and durable treatment for tracheomalacia. While most patients improve early, further improvement may occur over time. Caution is warranted when considering aortopexy in children with underlying systemic syndromes.

## Introduction

Tracheomalacia is characterised by the partial or complete collapse of the tracheal wall, particularly during expiration [1]. This leads to airway occlusion and variable clinical presentation, ranging from a ‘barking’ cough through exertional dyspnoea to acute episodes of cyanosis and acute life-threatening episodes (ALTEs). While tracheomalacia may occur in isolation, it is frequently associated with congenital anomalies such as oesophageal atresia, with or without tracheoesophageal fistula [2, 3]. In most cases, the condition is self-limiting and requires no intervention, but where symptoms are severe, surgical intervention may be warranted.

Aortopexy, first described by Gross and Newhauser [4], is the most common surgical procedure to address tracheomalacia. This entails the anterior repositioning of the aorta which, due to the shared fascial investments, relieves pressure on the trachea, ensuring airway patency during respiration. Various surgical approaches to aortopexy have been described including sternotomy [5], anterior mediastinotomy [6], thoracotomy [7], thoracoscopy [8] and, less commonly, a suprasternal incision [9].

While aortopexy is recognised to be effective, with published success rates of approximately 80% [10–13], its long-term outcomes remain poorly reported. Moreover, there is limited consensus regarding its indications and the specific patient populations most likely to benefit.

We aimed to determine the long-term outcomes of patients with tracheomalacia treated with aortopexy via a suprasternal incision to better understand the predictors for operative success or failure and to further define indications for aortopexy.

## Methods

All patients undergoing aortopexy performed by a single surgeon between February 2016 and May 2019 were included. These patients were referred through a multidisciplinary team (MDT) pathway including paediatric ENT, general and cardiac surgeons, cardiologists, paediatric intensive care, and respiratory physicians. Preoperative evaluation followed a standardised workup, including inspiratory and expiratory computed tomography (CT) and dynamic flexible bronchoscopy (DFB), to assess the severity and extent of tracheomalacia.

### Operative Technique

Aortopexy was performed via a suprasternal skin crease incision using a standardised anterior cervical approach. Following division of the platysma and splitting of the strap muscles, thymic tissue was reduced as required to facilitate access to the anterior mediastinum. This allowed exposure of the brachiocephalic vein and subsequent identification of the trachea and aortic arch at the level of the origin of the innominate artery.

Dissection was undertaken to achieve safe exposure of the relevant mediastinal structures while minimising dissection to the natural facial attachments between the trachea and vessels. When additional exposure was required, a limited manubrial split was performed.

Suspension was achieved using two 5/0 polypropylene pledgeted sutures placed in the anterior wall of the aorta at the origin of the innominate artery. The sutures were passed through the manubrium using an epidural needle and secured to achieve anterior elevation of the whole aortic arch complex, resulting in indirect anterior displacement of the trachea via its fascial attachments. Intra-operative flexible bronchoscopy was used to confirm adequacy and stability of tracheal opening, with additional tracheopexy sutures performed if required.

### Outcomes

The short-term outcomes of the majority of this cohort following aortopexy have been previously reported [14]. This study represents an extended longitudinal follow-up of the same cohort, with the inclusion of additional patients treated since the original publication, all of whom were managed using a consistent operative technique and follow-up protocol.

Electronic patient records (EPR) were reviewed and data including patient demographics, medical and surgical history, preoperative symptoms, and pre- and postoperative DFB findings were extracted. Patients were grouped according to underlying pathology to facilitate assessment of risk profiles [15]. Outcome classification was defined as ‘successful’ if there was partial or complete resolution of symptoms and ALTEs, and ‘unsuccessful’ if symptoms were unchanged postoperatively, or if redo aortopexy or tracheostomy was required.

Short-term outcomes were defined as those recorded at first postoperative clinical follow-up, and long-term outcomes as those documented at the last recorded clinical contact. Data are presented as medians with interquartile ranges (IQR), unless otherwise specified.

The study was registered with the institutional clinical audit department as service evaluation (Reference 64103).

## Results

In the time period studied, a total of 28 paediatric patients (male *n* = 22, 78%) underwent suprasternal aortopexy. Median age at surgery was 8 months (range 19 days − 5 years and 9 months). Underlying conditions lay in four categories: OA/TOF (*n* = 8), premature birth (*n* = 10), syndromic (*n* = 8; Prader Willi, Leri-Weill, Rubinstein Taybi, Shwachman-Diamond, and Russel Silver syndromes, and 22q11.2 duplication syndromes), and primary tracheomalacia (*n* = 7) where there was no other significant medical history.

### Baseline Patient Demographics

**Table 1:** Baseline characteristics, presenting symptoms and demographics

*Characteristics*	*Patient Group*			
	**OA/TOF** (*n* = 8)	**Prematurity** (*n* = 6)	**Syndromic** (*n* = 7)	**Primary** (*n* = 7)
*Baseline*				
Female sex (%)	3 (37.5)	0 (0)	2 (28.57)	1 (14.29)
Age at Surgery [months (median and IQR)]	6.5 (28.38)	19 (18.5)	3.5 (13.5)	6 (0)
*Presenting Symptoms (%)*				
Respiratory distress	6 (75)	4 (66.67)	4 (57.14)	6 (85.71)
Stridor	4 (50)	4 (66.67)	5 (72.43)	3 (42.86)
Recurrent infections	2 (25)	2 (33.33)	0 (0)	1 (14.29)
Cyanosis	0 (0)	1 (16.67)	1 (14.29)	4 (57.14)
Cough	4 (50)	3 (50)	0 (0)	0 (0)
Swallowing/feeding difficulty	1 (12.5)	0 (0)	3 (42.86)	2 (28.57)
*Site of Malacia (%)*				
Proximal to mid-thirds	4 (50)	2 (33.33)	3 (42.86)	1 (14.29)
Mid-third	4 (50)	3 (50)	2 (28.57)	2 (28.57)
Mid to distal thirds	2 (25)	0 (0)	1 (14.29)	0 (0)
Distal third	0 (0)	3 (50)	1 (14.29)	5 (71.43)
*Operative Technique (%)*				
Tracheopexy Performed	5 (62.5)	1 (16.67)	0 (0)	2 (28.57)

Table displays the baseline characteristics, presenting symptoms, anatomical distribution of malacia, and operative techniques. All values are given as *n* (%) unless stated otherwise. *OA/TOF:* Oesophageal atresia/tracheoesophageal fistula

The median age at surgery varied across patient groups, with syndromic patients undergoing surgery at the youngest median age of 3.5 months (*IQR* 13.5 months), while premature patients had the oldest median age at 19 months (*IQR* 18.5 months) (Table 1).

Respiratory distress was the most common presenting symptom, reported in 71.43% of the cohort (*n* = 20), and was most prevalent in the “other” group (85.71%, *n* = 6). Stridor was observed in 57.14% of patients (*n* = 16), with the highest incidence in the syndromic group (71.43%, *n* = 5). Pre-operative recurrent infections were reported in 14.29% of patients overall (*n* = 3), with the highest prevalence in the premature group (33.33%, *n* = 2).

Tracheopexy was performed in 28.57% of the cohort (*n* = 8), with the highest frequency in the OA/TOF group (62.5%, *n* = 5).

### Short and Long-term Outcomes

One patient in the ‘primary’ group died during the long-term follow up period, there was otherwise full follow up to a median of 6 years and 7 months (range 5 years and 2 months − 8 years and 9 months).

**Fig. 1:**
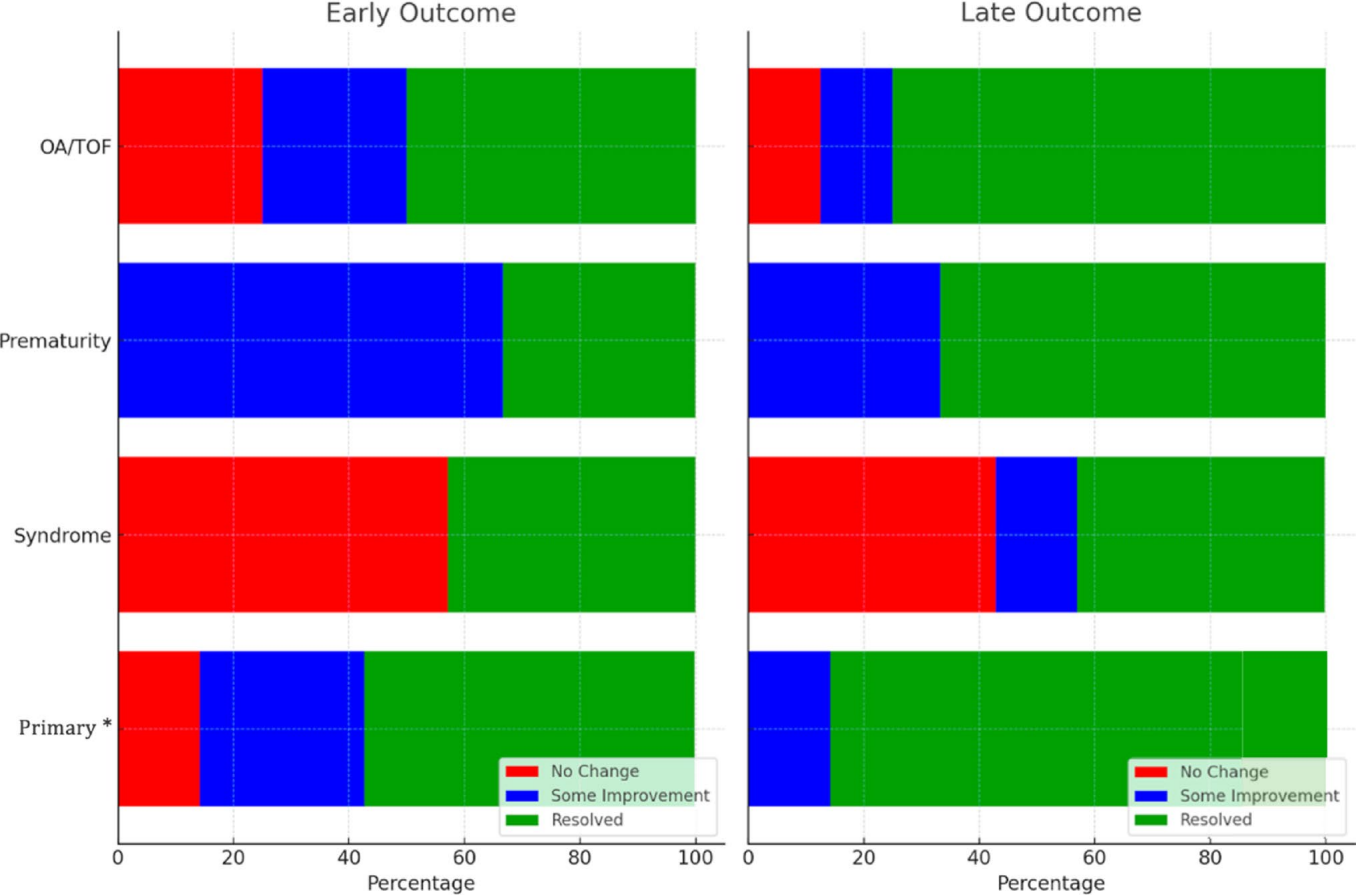
Short and Long-Term Outcomes by Category. Proportion of patients in each group achieving no change in symptoms, some improvement, and complete symptom resolution at early and late follow-up. *Red* represents the proportion with no change, *blue* some improvement and *green* full resolved. *OA/TOF*, oesophageal atresia with tracheoesophageal fistula; * = one patient died before long-term review

### Symptom Improvement Following Suprasternal Aortopexy

Long-term follow-up revealed a clear trend toward improvement or resolution of symptoms across all groups. At short-term follow-up, 13 patients (46%) achieved complete symptom resolution (Fig. 1). By long-term follow-up, this increased to 18 patients (64%). A total of 23 patients (82%) achieved a ‘successful’ clinical outcome, defined as either complete or partial symptom resolution, by long-term follow-up.

### Outcomes by Group

At short-term follow-up, 50% of patients with OA/TOF (*n* = 4) achieved complete resolution of symptoms, which increased to 75% (*n* = 6) in the long-term. There was also a decrease in the number of patients with no change in symptoms from 25% (*n* = 2) to 12.5% (*n* = 1).

Premature patients demonstrated the most consistent initial outcomes, with all individuals in this group achieving a ‘successful aortopexy’ at short-term follow-up. This success was sustained at long-term follow-up, with a shift from the majority of patients showing partial improvement in the short term to complete resolution of symptoms by long-term follow-up. In the short term, one-third of patients (*n* = 2) achieved complete symptom resolution, while two-thirds (*n* = 4) demonstrated partial improvement. By long-term follow-up, the proportion of patients achieving complete resolution increased to two-thirds (*n* = 4), with the remaining one-third (*n* = 2) showing partial improvement.

Patients with syndromic conditions demonstrated the least favourable outcomes, with consistently lower rates of ‘successful’ resolution at both short- and long-term follow-up compared to the other groups. The percentage of patients achieving complete resolution remained unchanged at 42.8% (*n* = 4) and limited improvement was observed in only one patient (14%).

Lastly, the primary group also demonstrated some improvement over time. Although the overall number of patients with a ‘successful aortopexy’ remained the same at both short-term and long-term follow-up, the proportion of patients achieving complete symptom resolution increased from 57% (*n* = 4) at short-term follow-up to 83% (*n* = 5) at long-term follow-up.

## Discussion

We present the long-term outcomes of a large case series of children with tracheomalacia treated with suprasternal aortopexy. This study builds upon our previously published cohort by reporting extended follow-up within an expanded and consistently managed series, allowing a more comprehensive assessment of outcomes. Our results support the efficacy and durability of this surgical technique, with symptomatic improvement in 75% of patients in the immediate postoperative period, and 82% exhibiting sustained improvement of symptoms at extended follow-up assessment.

Existing literature presents an overall success rate of approximately 80% for aortopexy in addressing tracheomalacia, with a reported redo rate of roughly 1% [14].

### Efficacy of Suprasternal Aortopexy

The most favourable outcomes in our cohort were observed in children with OA/TOF, prematurity, and primary malacia. This is in keeping with Dave and Currie, who analysed a cohort of 28 patients presenting with oesophageal atresia or primary tracheomalacia [7]. They report symptom resolution in 92% of cases. In our cohort, aortopexy was performed in syndromic patients only when DFB clearly identified tracheomalacia. Despite this, outcomes following aortopexy in this group were poor. This suboptimal response likely reflects the multifactorial nature of their conditions [16] and has been reported previously [10]. The report of a large cohort (*n* = 73) of patients undergoing aortopexy presented favourable outcomes in cases associated with OA/TOF, vascular compression, or primary tracheomalacia [15]. Conversely, patients with severe cardiac anomalies or significant comorbidities did worse, with higher rates of secondary interventions (redo surgeries, tracheostomy, or stenting) as well as prolonged intensive care unit admissions. The anatomical variations, diminished physiological reserves, and more extensive airway involvement commonly seen in syndromic patients likely contribute to the reduced success of aortopexy in this group.

### Ongoing Symptoms

In our cohort, ALTEs were effectively resolved following aortopexy; however, milder persistent symptoms, such as abnormal cough or stridor, continued in some patients. This is similar to existing reports where no ALTEs or apnoeas were observed following surgery although more minor symptoms did persist [7, 16, 17]. The commonest ongoing symptom in our cohort was recurrent chest infection, this is in common with several previous aortopexy cohorts [14] with various surgical approaches [13, 18–20], with many patients remaining prone to infections despite the resolution of respiratory distress, particularly within the first decade post-surgery [21]. The underlying cause of this increased susceptibility, whether attributable to the aortopexy procedure itself or a residual symptom of the original pathology, remains uncertain.

Long-term outcomes following aortopexy remain underreported in the literature, with most case series small or limited in the duration of their follow-up [5–8, 10]. However, late improvements in symptoms have been described with one report finding that 73% of their 30-patient cohort were asymptomatic at long-term follow-up [22]. Our series supports the possibility of late symptom resolution, with several patients demonstrating gradual, sustained improvements over extended follow-up periods. This is mostly likely due to patients following the known natural history of tracheomalacia where symptoms commonly resolve after the first few years of life, particularly considering the higher rate of later improvement in the premature and primary malacia group.

### Comparison with Other Surgical Approaches

Currently, no single surgical approach for aortopexy has been shown to be superior [10]. Choice of technique is currently largely determined by the surgeon’s preference. Among the described techniques, left anterior thoracotomy is the most commonly employed, although alternative approaches, such as median sternotomy, anterior mediastinotomy, and thoracoscopy, are utilised in roughly one-third of patients [10]. A large observational cohort study comparing left thoracotomy with median sternotomy found no significant difference in the efficacy of aortopexy between these two approaches [15]. However, thoracotomy, particularly when performed in patients with congenital cardiac deformity, has been associated with long-term scoliosis [23], suggesting the potential for complications with more invasive approaches.

Theoretically, minimally invasive thoracoscopy offers potential advantages, including reduced postoperative pain and morbidity, in comparison to thoracotomy or sternotomy [24]. However, the long-term durability of thoracoscopic procedures is inconsistent, with studies showing that open thoracotomy and partial sternotomy typically yield more reliable, lasting results. Jennings et al. observed a 38% recurrence rate in thoracoscopic cases, compared to no recurrences in patients undergoing partial sternotomy or open thoracotomy [25]. An anterior mediastinal approach via a muscle splitting supra-sternal creates space within the anterior mediastinum, enabling anterior elevation of the aorta and accommodating additional procedures such as tracheopexy when necessary. At the same time, it preserves sternal integrity and minimises other surgical trauma, enhances cosmetic outcomes, and may offer advantages such as faster recovery times and improved preservation of postoperative respiratory mechanics [6, 26]. Within this context, the suprasternal approach emerges as a promising alternative that may offer a balance between the benefits of minimally invasive procedures and the long-term durability of open surgery.

### Limitations and Future Directions

A notable strength of this study lies in its extended follow-up period, with a median duration of 6 years and 7 months, and a minimum follow-up of 5 years. This prolonged follow-up provides a better understanding of the efficacy and long-term durability of suprasternal aortopexy, allowing for the identification of both successes and failures that might not have been apparent in shorter-term evaluations. Given the limited reports on long-term outcomes of aortopexy, this extended observation contributes to the existing body of literature. Furthermore, the completeness of the follow-up data, with no patients lost to follow-up, ensures the robustness and reliability of our findings. However, it is important to recognise the potential limitations inherent in the retrospective design of the study. It is also a single-institution study, with all procedures conducted by a single surgeon. While this may contribute to a high degree of consistency in surgical technique, it inherently limits the external validity and generalisability of our findings. Variations in surgical expertise, postoperative care protocols, and patient demographics across different institutions will result in varying outcomes. Additionally, although our cohort is the largest reported to date for suprasternal aortopexy, the absolute number of participants remains small, particularly when stratified by age or comorbidity. Additionally, the scope of our study is somewhat limited due to the selective nature of the patient cohort. Not all patients referred for aortopexy were included in our analysis, and the outcomes of those who did not undergo aortopexy are not known. This gap warrants further exploration, particularly considering the natural history of tracheomalacia, which often exhibits gradual improvement over time.

Determining the optimal approach to the surgical management of tracheomalacia will require large scale, prospective studies. Given the relative rarity of the condition and intervention, this will need to be multi-centre and collaborative in their design. Comparisons need to be made not just between different surgical approaches but also between surgical and conservative management of tracheomalacia. This will help define both thresholds for intervention and the ideal approach. It seems unlikely that a randomised controlled trial would be feasible, prospective observational studies would provide valuable insights.

## Conclusion

Suprasternal aortopexy is a safe and effective treatment for tracheomalacia, providing durable symptom relief. The best outcomes are seen in patients with oesophageal atresia/tracheoesophageal fistula and those born prematurely. While most patients demonstrate immediate postoperative improvement, a significant proportion experienced further symptom improvement, with the majority achieving complete resolution at long-term follow-up. Deterioration after initial symptom improvement was not observed. However, caution should be exercised when considering this procedure for children with complex systemic syndromes, as these patients often exhibited very limited improvements.

The suprasternal approach to aortopexy appears to offer comparable efficacy to other open surgical techniques, at the same time, combined with the benefits of less invasive approaches. Further study is needed but it may prove to be the optimal surgical approach for aortopexy in childhood.

## Data Availability

Data Availability: The datasets generated and analysed during this study are available from the corresponding author upon reasonable request. Access may be subject to institutional and ethical approval to ensure patient confidentiality and compliance with data protection regulations.
